# Underwater Target Detection Utilizing Polarization Image Fusion Algorithm Based on Unsupervised Learning and Attention Mechanism

**DOI:** 10.3390/s23125594

**Published:** 2023-06-15

**Authors:** Haoyuan Cheng, Deqing Zhang, Jinchi Zhu, Hao Yu, Jinkui Chu

**Affiliations:** 1 College of Engineering, Ocean University of China, Qingdao 266100, China; zhangdeqing@stu.ouc.edu.cn (D.Z.); zjc@ouc.edu.cn (J.Z.); 2 Key Laboratory for Micro/Nano Technology and System of Liaoning Province, Dalian University of Technology, Dalian 116024, China; 201664058@mail.dlut.edu.cn (H.Y.); chujk@dlut.edu.cn (J.C.)

**Keywords:** underwater target detection, image fusion, unsupervised learning, attention mechanism, polarization

## Abstract

Since light propagation in water bodies is subject to absorption and scattering effects, underwater images using only conventional intensity cameras will suffer from low brightness, blurred images, and loss of details. In this paper, a deep fusion network is applied to underwater polarization images; that is, the underwater polarization images are fused with intensity images using the deep learning method. To construct a training dataset, we establish an experimental setup to obtain underwater polarization images and perform appropriate transformations to expand the dataset. Next, an end-to-end learning framework based on unsupervised learning and guided by an attention mechanism is constructed for fusing polarization and light intensity images. The loss function and weight parameters are elaborated. The produced dataset is used to train the network under different loss weight parameters, and the fused images are evaluated based on different image evaluation metrics. The results show that the fused underwater images are more detailed. Compared with light intensity images, the information entropy and standard deviation of the proposed method increase by 24.48% and 139%. The image processing results are better than other fusion-based methods. In addition, the improved U-net network structure is used to extract features for image segmentation. The results show that the target segmentation based on the proposed method is feasible under turbid water. The proposed method does not require manual adjustment of weight parameters, has faster operation speed, and has strong robustness and self-adaptability, which is important for research in vision fields, such as ocean detection and underwater target recognition.

## 1. Introduction

The ocean covers more than 70% of the earth’s total area. The marine ecosystem is one of the most productive and dynamic ecosystems on earth. Many scholars have conducted research in marine resource exploration, biological investigation, underwater vehicle navigation, and other fields [1,2,3,4]. Underwater optical images are one of the important media for exploring the ocean at present. However, due to the influence of a large number of floating particles in the water, the actual underwater images are seriously degraded, with problems such as high background noise, low contrast, and loss of details [1]. Therefore, the research of underwater image enhancement technology is of great significance and value for ocean exploration.

Researchers have shown that underwater polarization imaging technology can reduce the influence of backscattered light on underwater imaging to a certain extent by using the polarization characteristics of scattered light [5,6]. The degree of linear polarization (DoLP) images is used to characterize polarization characteristics and provide detailed features, which provide complementary scene information from different aspects. In order to obtain more complete information about the same scene, the intensity and DoLP images need to be fused. In the past decade, many image fusion methods have emerged in the field of image processing. Image fusion methods can be divided into different levels according to the fusion process: pixel level, feature level, and decision level. Among them, pixel-level image fusion has the most research and application. It can be divided into fusion methods based on the transform domain (image pyramid [7], wavelet transform [8], Ridgelets [9], curvelet transform [10]) and fusion methods based on the spatial domain (HIS transform [11], principal component analysis inverse transform [12], pixel value weighting, statistical model). These methods can realize multisource image fusion from the pixel level. The information on the fused image is more comprehensive, accurate, and reliable.

In recent years, deep learning technology has developed rapidly and made great breakthroughs in many problems in the field of computer vision and image processing. At present, there is more and more research on image fusion technology based on deep learning in digital imaging (multifocus image fusion [13], multiple exposure image fusion [14], etc.), multimodal imaging (visible light and infrared image fusion [15], medicine multimodal image fusion [16], polarization image fusion [17], etc.), remote sensing imaging [18] (multispectral and panchromatic image fusion, multispectral and hyperspectral image fusion, etc.), and other directions. Compared with the traditional methods, which have the limited ability to characterize the complex mapping relationship between the input image and the target image, the deep learning model has a strong ability to characterize the complex relationship between different signals. It can automatically extract the most effective features from the data to solve the difficulties of manual design. However, the polarization image fusion method based on deep learning has not been reported so far, which is one of the main contributions of this paper.

In this paper, the fusion network is applied to underwater polarization image processing, and a network model guided by unsupervised learning and attention mechanism is proposed. It is mainly divided into three modules: feature extraction, feature fusion, and image reconstruction. The feature extraction module incorporates an attention mechanism. The relevant loss function and weight parameters are constructed. The experimental results show that the proposed method can effectively fuse underwater light intensity and polarization image information and improve the quality of visual imaging. Compared with other traditional fusion algorithms, the proposed method has strong robustness and adaptability, which is of great significance for the application research of marine detection, underwater target recognition, and other visual fields.

## 2. Underwater Polarization Imaging

### 2.1. Underwater Imaging Model

**Figure 1:**
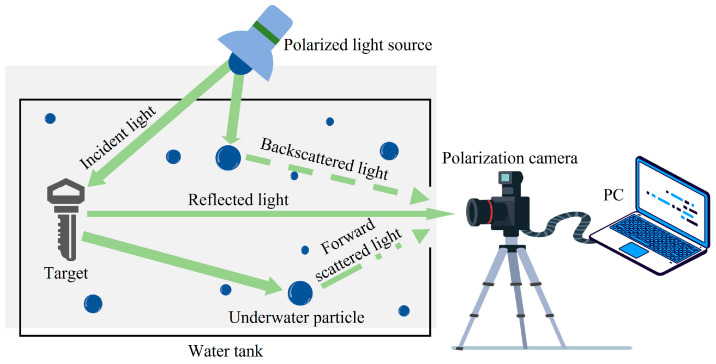
Underwater imaging model.

The Jaffe–McGlamery model [19,20] is one of the commonly used underwater imaging models, as shown in Figure 1. Many underwater image restoration algorithms are proposed based on this model. The Jaffe–McGlamery model states that the final image *I*(*x*, *y*) received by the detector is a linear combination of three components: the target reflected light *S*(*x*, *y*) received by the detector, the backscattered light *B*(*x*, *y*) scattered by the water body before the light source reaches the target, and the forward scattered light *F*(*x*, *y*) scattered by part of the target reflected light reaching the detector through the water body. The image can be expressed as

(1) I(x,y)=S(x,y)+B(x,y)+F(x,y).

The initial irradiance of the target is assumed to be *J*(*x*, *y*), and part of the energy is lost due to scattering and absorption when the light propagates from the target to the detector. Therefore, the reflected light *S*(*x*, *y*) of the target can be expressed as

(2) S(x,y)=J(x,y)⋅t(x,y),

*t*(*x*, *y*) is the transmittance of the medium, which is determined by the attenuation coefficient β(x,y) and the propagation distance ρ(x,y). In a single uniform medium, the attenuation coefficient β(x,y) is invariant in space, so $\beta (x,y)=\beta_{0}$. The propagation distance refers to the underwater part of the optical path between the object and the camera:

(3) $$t(x,y)=e^{-\beta (x,y)\rho (x,y)}.$$

Backscattered light *B*(*x*, *y*) is the background light scattered by water particles to the detector. It can be expressed as

(4) $$B(x,y)=B_{\infty }(1-t(x,y)),$$

where *B*_∞_ represents the underwater ambient light intensity at the infinite distance. Since the effect of forward scattering on imaging quality is minimal, its effect is usually ignored, so Equation (1) can be simplified as

(5) $$I(x,y)=J(x,y)\cdot t(x,y)+B_{\infty }(1-t(x,y)).$$

Thus, the initial irradiance *J*(*x*, *y*) of the object can be expressed as

(6) $$J(x,y)=\frac{I(x,y)-B_{\infty }(1-t(x,y))}{t(x,y)}.$$

### 2.2. Polarization Imaging Model

The Stokes vector method is one of the most commonly used polarization characterization methods in the field of polarization detection. This method can fully characterize the polarization characteristics of the light wave. The vector is composed of four parameters:

(7) $$S=\left[\begin{array}{l} I\\ Q\\ U\\ V \end{array}\right]=\left[\begin{array}{c} I_{0{}^{\circ}}+I_{90{}^{\circ}}\\ I_{0{}^{\circ}}-I_{90{}^{\circ}}\\ I_{45{}^{\circ}}-I_{135{}^{\circ}}\\ I_{r}-I_{l} \end{array}\right].$$

*S* represents the Stokes vector, which is a 4 × 1 column vector composed of four parameters: *I*, *Q*, *U*, and *V*. *I* represents the total light intensity received by the detector, *Q* represents the light intensity difference between the 0° and 90° polarization components *I*_0°_ and *I*_90°_, U represents the light intensity difference between the 45° and 135° polarization components *I*_45°_ and *I*_135°_, and *V* represents the intensity difference between the left- and right-handed circularly polarized components *I*_r_ and *I*_l_. The emergent light *S*′ = [*I*′, *Q*′, *U*′, *V*′]*^T^* can be obtained by the Mueller matrix:

(8) $$S^{\prime }=\left[\begin{array}{l} I^{\prime }\\ Q^{\prime }\\ U^{\prime }\\ V^{\prime } \end{array}\right]=\frac{1}{2}\left[\begin{array}{cccc} 1 & \cos2\theta & \sin2\theta & 0\\ \cos2\theta & \cos^{2}2\theta & \cos2\theta \sin2\theta & 0\\ \sin2\theta & \cos2\theta \sin2\theta & \sin^{2}2\theta & 0\\ 0 & 0 & 0 & 0 \end{array}\right]\left[\begin{array}{l} I\\ Q\\ U\\ V \end{array}\right].$$

*θ* is the included angle between the main optical axis and the reference line. *S*′ represents the outgoing light with angle *θ*. According to Equation (8), the intensity of the outgoing light with angle *θ* can be obtained as follows:

(9) $$I^{\prime }\left(\theta \right)=\frac{1}{2}\left(I+Q\cos2\theta +U\sin2\theta \right).$$

**Figure 2:**
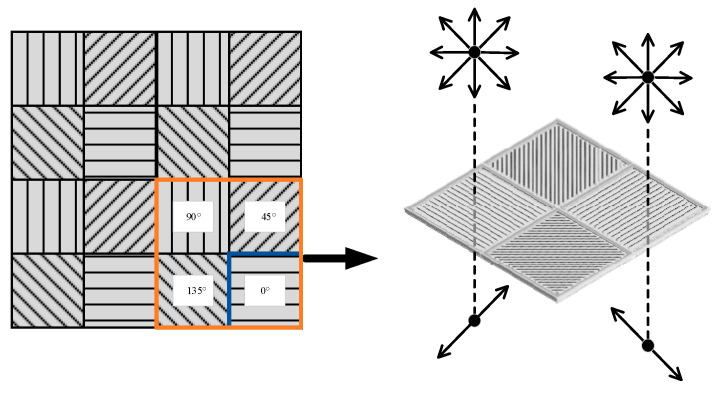
Polarization imaging model.

The polarization camera can obtain the light intensity image of the polarization directions of 0°, 45°, 90°, and 135° because each pixel of the CMOS sensor is placed with four polarizers of different angles (0°, 45°, 90°, and 135°), as shown in Figure 2. Every four pixels is set as a computing unit. Then, the light intensity of 0°, 45°, 90°, and 135° and the Stokes vector of the light can be obtained simultaneously. The Stokes vector can be used to further calculate the DoLP and angle of polarization of the incident light:

(10) $$DoLP=\frac{\sqrt{Q^{2}+U^{2}}}{I},$$

(11) $$\varphi =\frac{1}{2}\arctan\left(\frac{U}{Q}\right).$$

DoLP represents the proportion of linearly polarized components in the total light intensity. The angle of polarization refers to the dominant polarization direction of the incident light.

## 3. Deep Learning Method

### 3.1. Network Architecture

**Figure 3:**
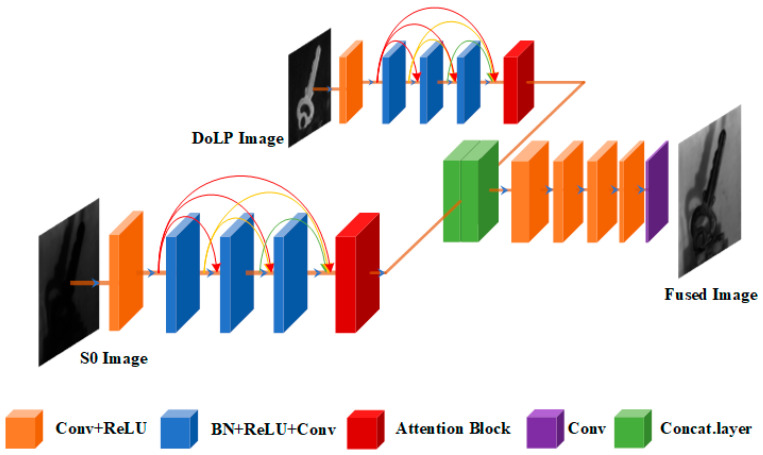
The network structure.

The network structure adopted in this paper is shown in Figure 3, which mainly consists of three modules: feature extraction, feature fusion, and image reconstruction. First, in the feature extraction module, the light intensity image and polarization image are input through two channels. The first layer is the convolution layer containing the 3 × 3 convolution kernel and activation function ReLU (rectified linear unit), which is used to extract low-level features. The second layer is the DenseBlock module containing 3 convolution layers to extract high-level features, in which each convolution layer also uses a 3 × 3 convolution kernel. The operation step of the convolution kernel is 1. Before the convolution operation, there are the BN (batch normalization) layer and ReLU activation function. This sort can speed up the training speed of the network. The two input channels of light intensity image and polarization image share the same weight, which can reduce the computational complexity of the network. This is followed by the attention unit (see Section 3.2), which takes the feature map of the previous layer as input. It is able to capture the global relationships in the data and guide the network to learn the distribution of the feature map. Second, in the feature fusion module, the feature map output by the feature extraction module is superimposed. The channel size of the two feature maps is 128, and the channel size of the fused feature map after being superimposed is 256. Finally, the output of the feature fusion module is used as the input of the image reconstruction module. The image reconstruction module includes 5 transposed convolution layers, and the convolution kernel size of each transposed convolution layer is also 3 × 3. The fusion results are reconstructed from the fusion features through these 5 transposed convolution layers. A more detailed network architecture is shown in Table 1.

### 3.2. Attention Mechanism

**Figure 4:**
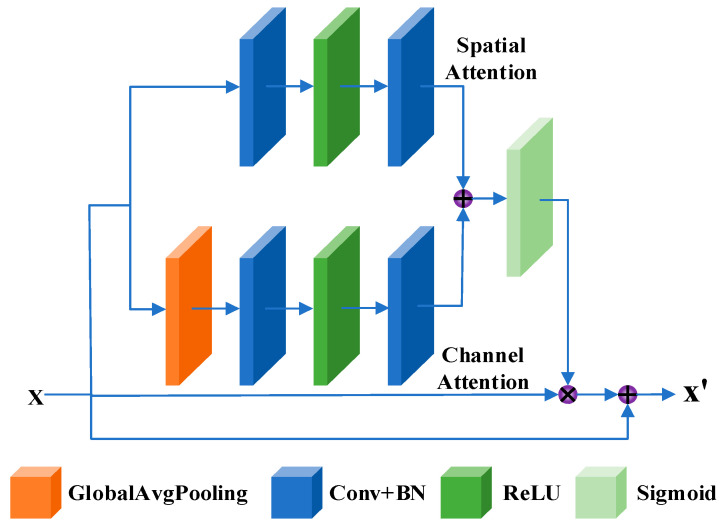
Attention unit structure.

The attention unit combines channel attention and spatial attention. Channel attention enables the network to learn the importance of features in the channel domain and give different weights to the feature map, so as to achieve the selective combination of light intensity image and polarization image in the channel domain. Spatial attention focuses on learning the effective information distribution of each layer of the feature map to improve the transmission of salient features. The attention unit includes a global mean pooling layer, a convolution layer, an activation layer, and a splicing layer, and its detailed structure is shown in Figure 4. Given $X\in R^{H\times W\times C}$ and $X^{\prime }\in R^{H\times W\times C}$ as the input and output of the attention unit, the calculation process of the attention unit is

(12) $$X^{\prime }=\sigma \left(F_{c}\left(X\right)⊕F_{s}\left(X\right)\right)⊗X+X,$$

where σ is the sigmoid activation function, *F_c_* is the channel attention branch, *F_s_* is the space attention branch, ⊕ is the broadcast addition operation, and ⊗ is the element-by-element multiplication operation.

When the input feature map $X\in R^{H\times W\times C}$ passes through the channel attention branch, the channel feature $X_{c}\in R^{1\times 1\times C}$ is obtained through the global average pooling layer, and then the channel feature size $1\times 1\times \frac{C}{r}$ obtained by point-by-point convolution of *PWConv*_1_, *BN* layer, and *ReLU* activation function. The channel attention feature map $X_{c}$ of size 1×1×C is obtained by point-by-point convolution of *PWConv*_2_ and *BN* layer. $F_{c}$ is expressed as

(13) $$F_{c}\left(X\right)=BN\left(PWConv_{2}\left(\delta \left(BN\left(PWConv_{1}\left(GAP\left(X\right)\right)\right)\right)\right)\right),$$

where δ is the *ReLU* activation function, and *GAP* is the global average pooling. Similar to the channel attention branch, when passing through the spatial attention branch, 3×3 convolution *Conv*_1_, *BN* layer, and *ReLU* activation function were first used to obtain the feature map of the size $H\times W\times \frac{C}{r}$. To obtain the spatial attention feature map of size H×W×C1×1, convolution *PWConv*_2_ and *BN* layer were used. $F_{s}$ can be expressed as:

(14) $$F_{s}\left(X\right)=BN\left(PWConv_{2}\left(\delta \left(BN\left(Conv_{1}\left(X\right)\right)\right)\right)\right).$$

### 3.3. Loss Function

The loss function in this paper adopts the globally weighted *SSIM* (structural similarity) loss function, which is a multiscale and weighted *SSIM* (*MSW* − *SSIM*) [17]:

(15) $$Loss^{MSW-SSIM}=1-\frac{1}{5}\cdot \sum_{\omega \in \left[3,5,7,9,11\right]}^{}\left(\begin{array}{l} \gamma_{\omega }\cdot Loss^{SSIM}\left(I_{S_{0}},I_{f};\omega \right)\\ +\left(1-\gamma_{\omega }\right)\cdot Loss^{SSIM}\left(I_{DoLP},I_{f};\omega \right) \end{array}\right).$$

${\mathrm{Loss}}^{\mathrm{SSIM}}\left(x,\;y;\;\omega \right)$ is a loss function based on the *SSIM*, which represents the structural similarity of the image x and y on window *ω*:

(16) $$Loss^{SSIM}\left(x,y;\omega \right)=\frac{\left(2{\bar{\omega }}_{x}{\bar{\omega }}_{y}+C_{1}\right)\left(2\sigma_{\omega_{x}\omega_{y}}+C_{2}\right)}{\left({\bar{\omega }}_{x}^{2}+{\bar{\omega }}_{y}^{2}+C_{1}\right)\left(\sigma_{\omega_{x}}^{2}+\sigma_{\omega_{y}}^{2}+C_{2}\right)}.$$

$\omega_{x}$ is the region of the image within the window ω, and ${\bar{\omega }}_{x}$ is the mean of $\omega_{x}$. The variables $\sigma_{\omega_{x}}^{2}$ and $\sigma_{\omega_{x}\omega_{y}}$ are the variance of $\omega_{x}$ and the covariance of $\omega_{x}$ and $\omega_{y}$, respectively. The remaining ones, $\omega_{y}$, ${\bar{\omega }}_{y}$, and $\sigma_{\omega_{y}}^{2}$, represent the corresponding meanings. $C_{1}$ and $C_{2}$ are constants to avoid instability when ${\bar{\omega }}_{x}+{\bar{\omega }}_{y}$ and $\sigma_{\omega_{x}}+\sigma_{\omega_{y}}$ are very close to zero, respectively.

The multiwindow *SSIM* is proposed in the loss function to solve the problem of image detail at different scales. The window sizes used include 3, 5, 7, 9, and 11. Different windows can extract features of different scales. In addition, ${\mathrm{Loss}}^{\mathrm{SSIM}}{(I}_{S_{0}}{,\;I}_{f};\;\omega )$ and ${\mathrm{Loss}}^{\mathrm{SSIM}}{(I}_{\mathrm{DoLP}}{,\;I}_{f};\;\omega )$ use the weight coefficient, which is based on $\sigma_{\omega_{S_{0}}}^{2}$ and $\sigma_{\omega_{DoLP}}^{2}$, defined as shown in Equation (17). When the window ω of the intensity image $S_{0}$ variance is greater than the corresponding DoLP image, it indicates that the local region of $S_{0}$ has more image details; that is, the weight coefficient $\gamma_{\omega }$ corresponding to the image of $S_{0}$ should be larger.

(17) $$\gamma_{\omega }=\frac{g\left(\sigma_{\omega_{S_{0}}}^{2}\right)}{g\left(\sigma_{\omega_{S_{0}}}^{2}\right)+g\left(\sigma_{\omega_{DoLP}}^{2}\right)}.$$

$\sigma_{\omega_{S_{0}}}^{2}$ is the variance of the intensity image $S_{0}$ within the window ω; $\sigma_{\omega_{DoLP}}^{2}$ is the variance of the *DoLP* image within the window ω. $g\left(x\right)=\max\left(x,\;0.0001\right)$ is a correction function to increase the robustness of the solution.

In addition, *MSW* − *SSIM* can retain high-frequency information, but it is insensitive to uniform deviation, which can easily lead to changes in brightness. Therefore, the integration of *MSW* − *SSIM* with the *L*_1_ norm loss function can ensure the brightness of fusion results. The *L*_1_ norm loss function can be expressed as

(18) $$Loss^{L_{1}}=\frac{1}{M\cdot N}{\left\|I_{avg}-I_{f}\right\|}_{1},$$

where *M* and *N* are the height and width of the image, respectively. $I_{avg}$ is the average value of $I_{S_{0}}$ and $I_{DoLP}$. $I_{f}$ is the fused image. Then the final loss function can be expressed as

(19) $$L^{Mix}=Loss^{MSW-SSIM}+\alpha \cdot Loss^{L_{1}}.$$

where α is a balance parameter.

## 4. Experiment

In order to obtain the dataset, the underwater imaging experiment was conducted. The experimental device and layout are shown in Figure 1, which mainly includes a polarization camera, glass water tank, polarization light source, and target object. The resolution of the polarizing camera (PHX0550S-P) is 2448 × 2048, and the number of bits is 12. It adopts focal-plane polarizing imaging and can take four images of linear polarized light intensity with polarization angles of 0°, 45°, 90°, and 135° at one time. The focal length of the lens is 10.5 mm. The polarized light source consists of an LED light source and a linear polarizer. A water tank (500 mm × 250 mm × 250 mm) was used as a container and the inside was covered with black flannel to avoid interference from ambient light. The target was placed in the water tank filled with water. The milk was prepared in the water tank to simulate the underwater situation with suspending particles. The light intensity and polarization images were obtained. Finally, a dataset containing 150 sets of images was constructed. Each set of images was composed of corresponding light intensity and polarization images, whose size was 1224 × 1024. A total of 100 groups were used as the training set, and the remaining 50 groups were used as the verification and test sets. In addition, the image of the dataset was flipped and trimmed to 80 × 80 as the input of network training. The training process was carried out on the server, whose graphics card was NVIDIA GeForce RTX 2080 Ti, whose processor was i9-9600X, and whose memory was 128 G. After weight initialization, the optimization was performed using the Adam optimizer with a minibatch size of 128. The learning rate was initially set to 0.0001 and decayed exponentially at a rate of 0.99, with a maximum epoch set to 200.

## 5. Results

### 5.1. Image Enhancement

Based on the above method, the network was trained and the performance of underwater image fusion was tested. Information entropy (IE), standard deviation (SD), mutual information (MI), and SSIM were adopted to measure the quality of the fusion image objectively. IE represents the average information amount of an image, as shown in Equation (20). The more information amount, the greater IE. Image fusion will result in the increase in image information, and IE can reflect the degree of change.

(20) $$H(X)=E[\log\frac{1}{p(a_{i})}]=-\sum_{i=1}^{n}p(a_{i})\log p(a_{i}).$$

*E* is the statistical average, and $p(a_{i})$ represents the probability of the gray value $a_{i}$ appearing.

*SD* refers to the dispersion degree of the image pixel gray value relative to the mean (μ). If the *SD* is larger, it indicates that the gray levels in the image are more dispersed and the image quality is better. The calculation formula is as follows:

(21) $$SD=\sqrt{\frac{1}{MN}\sum_{i=1}^{M}\sum_{j=1}^{N}{\left(I\left(x,y\right)-\mu \right)}^{2}},\mu =\frac{1}{MN}\sum_{i=1}^{M}\sum_{j=1}^{N}I\left(x,y\right).$$

*MI* can measure the degree of similarity between two images, that is, the amount of information of the original image obtained by the fusion of images. The larger the *MI* is, the more source image information is retained and the better the quality is. *MI* is based on the IE *H*(*A*) and the joint IE *H*(*A*,*B*) of the image:

(22) MI(A,B)=H(A)+H(B)−H(A,B).

The calculation formula adopted in this paper is

(23) $$MI=\frac{1}{2}MI(I_{S_{0}},I_{f})+\frac{1}{2}MI(I_{DoLP},I_{f}).$$

*SSIM* is a widely used image quality evaluation index. It is based on the assumption that structured information will be extracted when human eyes watch an image. The *SSIM* value ranges from −1 to 1. The closer it is to 1, the higher the similarity is and the better the fusion quality is. The calculation formula adopted in this paper is

(24) $$SSIM=\frac{1}{2}\cdot SSIM(I_{S_{0}},I_{f})+\frac{1}{2}\cdot SSIM(I_{DoLP},I_{f}).$$

**Figure 5:**
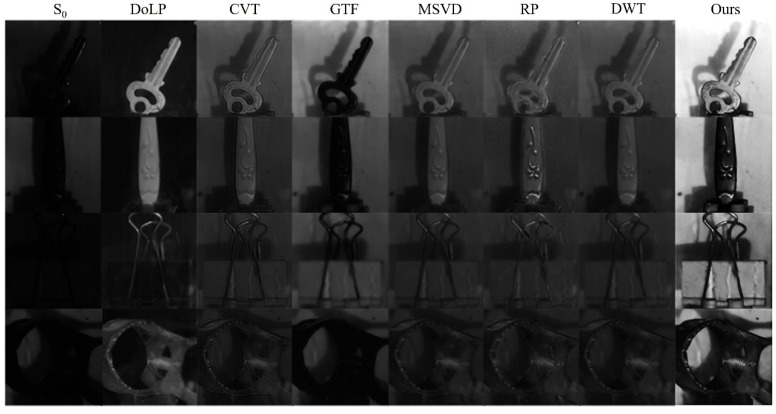
Comparison of fusion results of different methods for the partial test set.

The network training test results are shown in Figure 5. According to the image fusion results, it can be found that the quality of the light intensity image *S*_0_ is poor and the scene details are degraded seriously. However, after adding the polarization image for fusion, the target becomes clearer and the texture outline of the key can be clearly identified. According to the image index used for evaluation, the IE and SD after fusion increase by 24.48% and 139%, indicating that the proposed method can improve the quality of underwater images. In addition, the fusion image obtained by this method is compared with several other image fusion methods, including the curvelet transform (CVT) [10], the gradient transfer fusion (GTF) [21], the multiresolution singular value decomposition (MSVD) [22], the ratio of low-pass pyramid (RP) [7], and the discrete wavelet transform (DWT) [8]. As can be seen from Figure 5, the result of RP has poor visual quality. Artifacts are generated to a certain extent on the edge of the key and the shaded part, and there are more noises. The results of CVT, DWT, and MSVD have a certain degree of graininess, and the processing ability of shadows is poor. GTF results have high contrast, but the texture details of the key are not clear enough. However, the method presented in this paper has a relatively real visual effect without obvious artifacts and distortions and has a good processing effect on shadows. To further improve the image-enhancing effect, we also tested different network configurations, but the image quality is not improved much and the corresponding configurations are not valuable. Thus, we choose to only show the current configuration of the network, which already meets the actual requirements.

In order to objectively evaluate the performance of the method, the four image evaluation indexes introduced previously were used to evaluate the images in the test set, and the final results were averaged, as shown in Table 2. The method has better performance in the image evaluation indexes of IE, SD, MI, and SSIM, which further proves the effectiveness of the method.

Finally, the running time was evaluated on a server configured with NVIDIA GeForce RTX 2080 Ti, 3.1 GHz Intel Core i9-9600X, and 128 G RAM. The results are shown in Table 3. All methods were implemented using the average value of multiple groups of images in Python language. It can be seen that the processing speed of the proposed method is faster than other methods.

### 5.2. Target Segmentation

**Figure 6:**
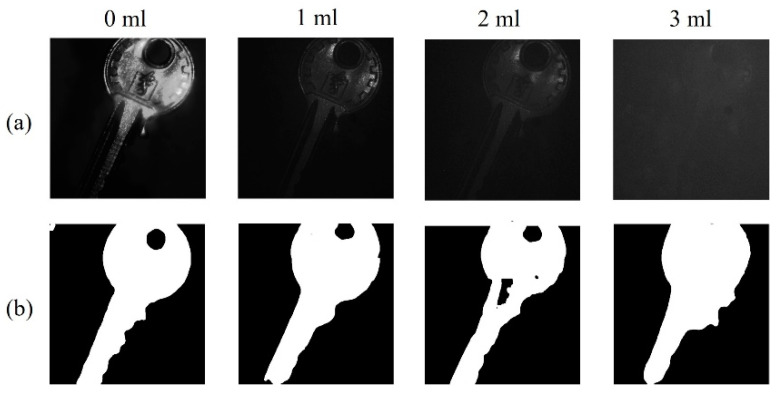
Results of target segmentation. (**a**) Original images. (**b**) Segmentation results.

Most of the existing underwater image segmentation algorithms are based on one of the light intensity information, spectral information, and polarization information, which has great limitations. It is necessary to make reasonable comprehensive use of light intensity and polarization information. In the study, we use the improved U-net network to extract features from the fusion image for image segmentation [23,24]. The framework and corresponding configuration of the image segmentation are similar to the image enhancement section of the previous part. We simulated different turbidities underwater by adding different volumes of milk (0, 1, 2, and 3 mL) into the water tank. Figure 6a,b are original images and segmentation results, respectively, at different water turbidities. With the increase in turbidity, the intensity of backscattered light increases, the clarity of the original image decreases, and the noise increases. When the milk volume is 3 mL, the image quality decreases obviously, but the general outline can still be detected in the segmentation results.

**Figure 7:**
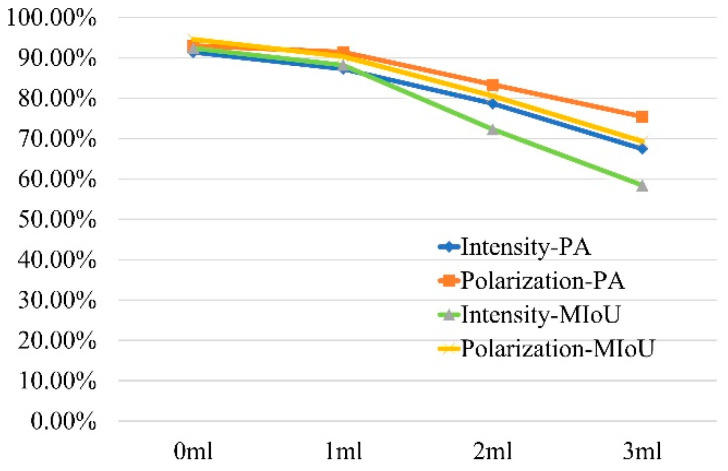
Quantitative analysis of target segmentation.

We used pixel accuracy (PA) and mean intersection over union (MIoU) to measure the accuracy of image segmentation. PA represents the ratio of correctly predicted pixels to all pixels in the image. The physical meaning of MIoU is that the overlap of the predicted and labeled region is divided by the combination of the predicted and labeled region. The evaluation index of target segmentation results with different water turbidities is shown in Figure 7. After adding polarization information, PA and MIoU are improved. The results show that the target segmentation based on the proposed method is feasible under turbid water.

## 6. Conclusions

Aiming at the problem of poor quality of underwater optical imaging, this paper proposes a method of applying a deep fusion network to underwater polarization images. By analyzing the underwater active polarization imaging model, we set up an experimental device to obtain the underwater polarization image and construct the training dataset. We establish an end-to-end network model based on unsupervised learning and attention mechanism guidance and design the loss function. The experimental results show that the method can improve the visual quality of the image and is superior to other methods. The processing speed of this method is faster than that of other methods, which shows the potential of the method to meet the requirements of real-time underwater video processing. Next, we improve the U-net network structure to extract features for image segmentation. The results show that the target segmentation based on the proposed method is feasible under turbid water. Future research includes building a more comprehensive dataset and improving the loss function and network module to further improve the quality of fusion image and meet the requirements of practical applications. At the same time, the processing efficiency of the algorithm will be improved to reduce the operation time and realize the real-time detection of underwater targets.

## Figures and Tables

**Table 1:** Network structure configuration.

Module	Input	Layer	Convolution Kernel Size	Input Channel	Output Channel	Activation Function	Output
Feature extraction	$S_{0}$	Conv	3 × 3	1	16	ReLU	
		Conv	3 × 3	16	16	ReLU	
		Conv Conv	3 × 3 3 × 3	32 48	16 16	ReLU ReLU	
		AttentionBlock	-	64	128	ReLU	$F_{S_{0}}$
	*DoLP*	Conv	3 × 3	1	16	ReLU	
		Conv	3 × 3	16	16	ReLU	
		Conv	3 × 3	32	16	ReLU	
		Conv	3 × 3	48	16	ReLU	
		AttentionBlock	-	64	128	ReLU	$F_{DoLP}$
Feature fusion	$F_{S_{0}},\;\;F_{DoLP}$	Concat	-	128 × 2	256	-	$F_{fused}$
Reconstruction	$F_{fused}$	Conv	3 × 3	256	128	ReLU	
		Conv	3 × 3	128	64	ReLU	
		Conv	3 × 3	64	32	ReLU	
		Conv	3 × 3	32	16	ReLU	
		Conv	3 × 3	16	1	-	$I_{f}$

**Table 2:** Quantitative results of polarization image fusion by different methods.

	CVT	GTF	MSVD	RP	DWT	Ours
IE	6.1123	6.9818	6.0591	6.4815	6.0834	7.1073
SD	17.87	42.62	17.11	21.93	18.21	40.46
MI	0.7374	1.2725	0.7574	0.36	1.4717	1.6808
SSIM	0.4953	0.3542	0.4959	0.2409	0.5049	0.5186

**Table 3:** Operating time of different methods.

	CVT	GTF	MSVD	RP	DWT	Ours
time/s	1.042	0.068	0.3568	0.082	0.5086	0.051

## Data Availability

Not applicable.
